# Prediction of WHO grade and methylation class of aggressive meningiomas: Extraction of diagnostic information from infrared spectroscopic data

**DOI:** 10.1093/noajnl/vdae082

**Published:** 2024-06-06

**Authors:** Roberta Galli, Franz Lehner, Sven Richter, Katrin Kirsche, Matthias Meinhardt, Tareq A Juratli, Achim Temme, Matthias Kirsch, Rolf Warta, Christel Herold-Mende, Franz L Ricklefs, Katrin Lamszus, Philipp Sievers, Felix Sahm, Ilker Y Eyüpoglu, Ortrud Uckermann

**Affiliations:** Faculty of Medicine, Medical Physics and Biomedical Engineering, Technische Universität Dresden, Dresden, Germany; Department of Neurosurgery, Faculty of Medicine and University Hospital Carl Gustav Carus, Technische Universität Dresden, Dresden, Germany; Department of Neurosurgery, Faculty of Medicine and University Hospital Carl Gustav Carus, Technische Universität Dresden, Dresden, Germany; Else Kroener Fresenius Center for Digital Health, Technische Universität Dresden, Dresden, Germany; Department of Neurosurgery, Faculty of Medicine and University Hospital Carl Gustav Carus, Technische Universität Dresden, Dresden, Germany; Faculty of Medicine, Department of Pathology, Technische Universität Dresden, Dresden, Germany; Department of Neurosurgery, Faculty of Medicine and University Hospital Carl Gustav Carus, Technische Universität Dresden, Dresden, Germany; Department of Neurosurgery, Faculty of Medicine and University Hospital Carl Gustav Carus, Technische Universität Dresden, Dresden, Germany; Division of Experimental Neurosurgery, Department of Neurosurgery, Heidelberg University, Im Neuenheimer Feld 400, 69120 Heidelberg, Germany; Division of Experimental Neurosurgery, Department of Neurosurgery, Heidelberg University, Im Neuenheimer Feld 400, 69120 Heidelberg, Germany; Laboratory for Brain Tumor Research, Department of Neurosurgery, University Medical Center Hamburg-Eppendorf, Hamburg, Germany; Laboratory for Brain Tumor Research, Department of Neurosurgery, University Medical Center Hamburg-Eppendorf, Hamburg, Germany; Department of Neuropathology, University Hospital Heidelberg, Heidelberg, Germany; CCU Neuropathology, German Consortium for Translational Cancer Research (DKTK), German Cancer Research Center (DKFZ), Heidelberg, Germany; Department of Neuropathology, University Hospital Heidelberg, Heidelberg, Germany; CCU Neuropathology, German Consortium for Translational Cancer Research (DKTK), German Cancer Research Center (DKFZ), Heidelberg, Germany; Department of Neurosurgery, Faculty of Medicine and University Hospital Carl Gustav Carus, Technische Universität Dresden, Dresden, Germany; Department of Neurosurgery, Faculty of Medicine and University Hospital Carl Gustav Carus, Technische Universität Dresden, Dresden, Germany; Else Kroener Fresenius Center for Digital Health, Technische Universität Dresden, Dresden, Germany; Department of Psychiatry and Psychotherapy, Division of Medical Biology, Faculty of Medicine and University Hospital Carl Gustav Carus, Technische Universität Dresden, Dresden, Germany

**Keywords:** infrared spectroscopy, meningioma, methylation class, neural network classifier, WHO CNS grade

## Abstract

**Background:**

Infrared (IR) spectroscopy allows intraoperative, optical brain tumor diagnosis. Here, we explored it as a translational technology for the identification of aggressive meningioma types according to both, the WHO CNS grading system and the methylation classes (MC).

**Methods:**

Frozen sections of 47 meningioma were examined by IR spectroscopic imaging and different classification approaches were compared to discern samples according to WHO grade or MC.

**Results:**

IR spectroscopic differences were more pronounced between WHO grade 2 and 3 than between MC intermediate and MC malignant, although similar spectral ranges were affected. Aggressive types of meningioma exhibited reduced bands of carbohydrates (at 1024 cm^−1^) and nucleic acids (at 1080 cm^−1^), along with increased bands of phospholipids (at 1240 and 1450 cm^−1^). While linear discriminant analysis was able to discern spectra of WHO grade 2 and 3 meningiomas (AUC 0.89), it failed for MC (AUC 0.66). However, neural network classifiers were effective for classification according to both WHO grade (AUC 0.91) and MC (AUC 0.83), resulting in the correct classification of 20/23 meningiomas of the test set.

**Conclusions:**

IR spectroscopy proved capable of extracting information about the malignancy of meningiomas, not only according to the WHO grade, but also for a diagnostic system based on molecular tumor characteristics. In future clinical use, physicians could assess the goodness of the classification by considering classification probabilities and cross-measurement validation. This might enhance the overall accuracy and clinical utility, reinforcing the potential of IR spectroscopy in advancing precision medicine for meningioma characterization.

## Lay Summary

Meningioma is a type of tumor that grows from the lining around the brain and spine. Some are benign and grow slowly, while others are aggressive and grow quickly. The aggressiveness of the tumor is typically determined after it is surgically removed and examined under a microscope or analyzed using genetic techniques, both of which take time. The authors of this study used infrared spectroscopy (IR), a technique that uses light to measure substances in a sample, to predict how aggressive the tumor is. They found that IR spectroscopy could predict the tumor’s aggressiveness with good accuracy, as confirmed by traditional pathology and genetic methods.

**Key Points**

- Aggressive types of meningioma have changed infrared (IR) spectroscopic signatures.
- Deep learning can predict WHO grade and methylation class of meningiomas exploiting IR spectra.

**Importance of the Study**

Meningiomas are the most prevalent primary brain tumors. With a minority exhibiting malignant behavior, early identification is crucial. Since IR spectroscopy offers intraoperative optical brain tumor diagnosis, we investigated the spectral signatures for the WHO CNS grading system and the methylation classes. While previous studies have delved into spectroscopy of meningiomas, our research is the first to include the rare anaplastic subtype. The identification of differences in the infrared spectra enabled the development of a translational approach for the detection of aggressive meningiomas. Importantly, neural network classifiers are needed for the identification of MC classes, presumably due to the complex and interacting processes associated with DNA methylation patterns. These findings may pave the way for intraoperative spectroscopic assessment of meningiomas to support clinical decision making in the pursuit for improved therapeutic strategies.

Vibrational spectroscopy includes infrared (IR) and Raman spectroscopy and has been suggested for brain tumor diagnosis. Many studies illustrate its enormous potential in neuro-oncology, demonstrating its ability to discriminate tumor from nontumor tissue, detect necrotic areas, reveal the tumor (sub)type,^1^ and identify the primary tumor of brain metastases.^2^ As these optical techniques are label-free and provide information about tissue immediately, they are envisioned as intraoperative tools for neurosurgery.^3–5^ In situ application of Raman spectroscopy during glioma surgery was already performed,^6^ while infrared spectroscopy of fresh brain tumor samples allowed successful brain tumor identification within minutes after resection.^7^ Thus, vibrational spectroscopy holds great promise for intraoperative applications to provide relevant information to aid clinicians in their decision making.

Meningiomas are the most frequent primary brain tumors. Most of them are nonmalignant, however a minority of meningiomas (0.7%) exhibits malignant behavior and with poor prognosis.^8^ They are graded as WHO CNS grade 1, WHO CNS grade 2, and WHO CNS grade 3 (anaplastic) based on histopathology and subtype according to current WHO classification.^9^ Several genetic aberrations and driver mutations have been identified in meningiomas, which endorsed for refinement of meningioma classification and grading. Brain tumors can also be classified based on their methylation profile.^10^ Methylome profiling stratifies meningiomas into benign (three subclasses), intermediate (two subclasses), and malignant. The methylation class (MC) correlates with progression and overall survival and might be superior to WHO grade for predicting patients at risk.^11^ Furthermore, recurrent meningiomas seem to arise from a genetically distinct subgroup.^12^ It is thus clinically important to identify those patients with aggressive meningiomas as early as possible in the clinical course in order to adapt the therapeutic regimen and follow-up care accordingly.

The spectroscopic signature of meningiomas has been previously investigated, however not for anaplastic meningioma WHO 3, as this is a rare subtype. The IR spectroscopic signatures of meningiomas WHO 1 were described on samples of four patients^13^ and analyses indicated reduced bands assigned to unsaturated fatty acids as main difference to brain tissue. Several studies focused on differences between nontumor brain tissue and several types of brain tumors (among those were meningiomas) and were successful in discriminating tissue types taking advantage of machine learning strategies.^14–16^ Other studies focused exclusively on meningioma with regard to neurosurgical applications. The analysis of frozen sections^17^ and fresh samples^18^ showed that vibrational spectroscopy is able to distinguish dura and meningioma tissue, as to be expected, mainly based on the spectral bands of collagen. Moreover, IR^19^ and Raman spectroscopy^20^ of dewaxed formalin-fixed paraffin-embedded tissue were able to discriminate meningiomas WHO 1 from meningiomas WHO 2 using principal component analysis (PCA) and discriminant analysis. Visible Resonance Raman spectroscopy confirmed this for fresh samples.^21^

Here, we have used infrared spectroscopy as a promising translational technology for the identification of aggressive meningiomas, based on both, the WHO classification and the methylation profile. Therefore, IR spectral differences were analyzed and strategies for predicting (i) WHO grades 2 or 3 and (ii) methylation classes intermediate or malignant were developed, taking into account the requirements for clinical use and evaluation of this information in the frame of patients’ therapy management.

## Materials and Methods

### Samples

Human brain tumor samples were obtained from routine surgical resection of brain tumors and one sample was investigated for each patient. Meningioma tumor samples from the Department of Neurosurgery, University Hospital Carl Gustav Carus at the TU Dresden (*n* = 38), from the Department of Neuropathology, University of Heidelberg (*n* = 4), and from the Department of Neurosurgery, University Medical Center Hamburg-Eppendorf, Hamburg (*n* = 5) were included. All meningiomas were diagnosed as WHO grade II or WHO grade III by the local neuropathologists; the diagnosis was confirmed and methylation class analysis^22^ was provided by the Department of Neuropathology, University of Heidelberg, Germany. Collection and use of tissue samples and data were done in accordance with local ethics regulations and approval.

Cryosections of 16 µm thickness were prepared on CaF_2_ slides (for infrared spectroscopy) and on glass slides (for reference histology).

### Infrared Spectroscopy

Infrared spectroscopy was conducted on frozen sections, as this is a validated approach for the investigation of biochemical signatures by vibrational spectroscopy. In future studies, this signature could then be transferred to analysis of fresh tissue or in situ. Three measurement positions being vital tumor and free of tissue preparation artifacts were selected on each sample in reference to a consecutive HE-stained tissue section; for 1 sample, only 2 suitable positions could be identified. Infrared spectra were acquired in transmission mode using a Vertex 70 FT-IR spectrometer with infrared microscope Hyperion 3000 (both Bruker Optik GmbH, Ettlingen, Germany) as described previously.^23^ The system is equipped with a focal plane MCT array detector in combination with a 15 × Cassegrain objective to deliver an array of 64 × 64 spectra over a 170 × 170 µm^2^ area at each measurement position. A new background spectrum was acquired prior to each measurement on CaF_2_ without sample. The spectral resolution was set to 6 cm^-1^ and 100 interferograms were collected, coadded and Fourier transformed by applying Blackman–Harris apodization and zero filling factor of 0, resulting in an acquisition time of 3 min. Each spectrum was ratioed to the background spectrum and the transmission spectra were converted to absorbance values.

To exclude contributions of residual water vapor and CO_2_ bands, an atmospheric compensation was calculated. A 4 × 4 spatial binning was performed to further improve the spectral quality and obtain 256 spectra for each measurement position, thus resulting in 768 spectra for each sample. FT-IR spectra were then reduced to the fingerprint region (950–1800 cm^-1^) and baseline corrected using a rubber band procedure (all performed in OPUS 7.2 software, Bruker Optic GmbH, Ettlingen, Germany). Spectra with artifacts were identified by visual inspection and excluded from further analysis.

### Classification of Spectral Datasets

All analyses were performed in MATLAB 2023a (The MathWorks Inc, Natick, MA, USA). The dataset was split into training (*n* = 18,345 spectra) and test set (n = 17,298 spectra) in a patient wise manner. Classifiers were developed and validated (leave-one-patient-out cross validation) on the training set and were afterwards applied to the independent test set. Data dimensionality reduction was performed in 3 different ways: (i) principal component analysis (PCA), using the MATLAB function *pca* and considering either the first five principal components (explaining 95% of variance) or the first 30 principal components (explaining 99.9% of variance); (ii) 4-fold spectral binning; (iii) manual feature selection (10 or 30 features) based on the Fisher coefficient. PCA and calculation of Fisher coefficients were performed on the training set.

The dataset was labeled according to either WHO CNS grade as “WHO 2” or “WHO 3” or according to methylation class intermediate “MC int” or malingnant “MC mal.” Classifiers were developed either based on linear discriminant analysis (MATLAB function *fitcdiscr*) or a fully connected neural network (MATLAB function *fitcnet* with 20 optimization steps *OptimizeHyperparameters auto*). The probability of class assignment was obtained for each spectrum of the test set. Subsequently, the probability of class assignment of each sample was calculated as the average value of the probabilities of all spectra belonging to the respective sample.

## Results

Samples of 47 meningioma cases were included in this study. Table 1 shows the diagnosis according to WHO grade and methylation class and the allocation of the samples to the test and training sets. In fact, many WHO 2 meningiomas belonged to the MC int and many WHO 3 samples belonged to MC mal. The grading of malignancy in the two different diagnostic systems was not concordant for a substantial number of samples.

**Table 1.** Histopathological Diagnosis of WHO CNS Grade and Methylation Class With Allocation to Training and Test set. One Sample was Investigated for Each Case. MC int: Intermediate, MC mal: Malignant

Diagnosis	Samples total	Samples training set	Samples test set
**WHO 2**	**21**	**11**	**10**
MC int	14	7	7
MC mal	7	4	3
**WHO 3**	**26**	**13**	**13**
MC int	10	5	5
MC mal	16	8	8
**Total**	**47**	**24**	**23**

**Figure 1.**
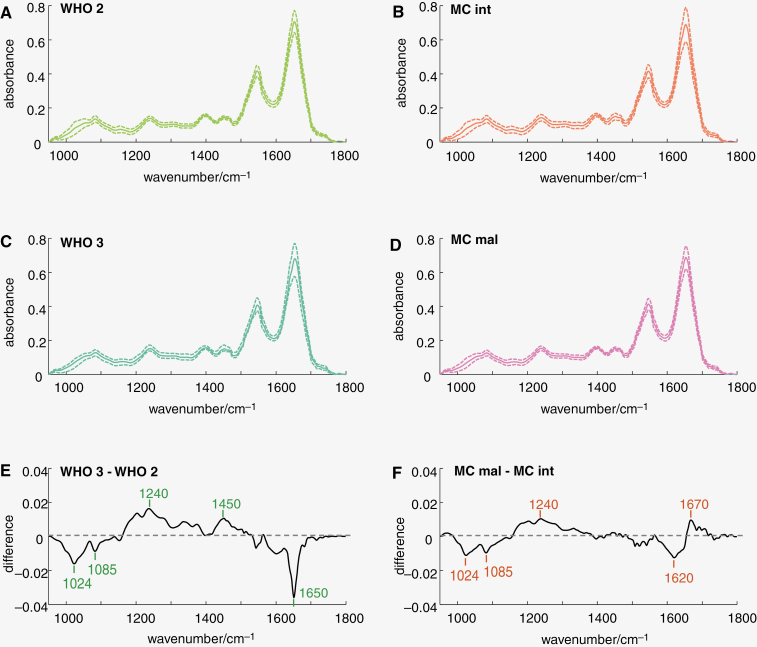
IR spectral signatures of meningioma. IR spectra of the training set were split according to WHO grade 2 versus WHO grade 3 (left) or methylation class intermediate versus malignant (right) and analyzed. A–D: Mean spectra (solid line) ± *SD* (dotted line) E, F: difference spectra as indicated

Three measurement positions were selected on a HE-stained tissue section and 256 IR spectra were obtained in a 16 × 16 array on consecutive unstained tissue sections at each position (Supplementary Figure 1). IR spectral signatures of meningiomas were analyzed on the training set (Figure 1). Mean spectra were calculated for the different WHO grades (left) or methylation classes (right). They all show the well-known IR spectral bands of brain tumors and look very similar on first sight. Besides, a similar standard deviation was observed for the different groups, indicating a similar variability (Figure 1A–D). The inspection of the difference spectrum showed reduced IR bands at 1024, 1085, and 1650 cm^-1^ and increased IR bands around 1240 and at 1450 cm^-1^ in meningiomas WHO 3 compared to meningiomas WHO 2 (Figure 1E). Spectral differences between methylation classes were found in similar spectral ranges but were smaller (Figure 1F). Here, reduced spectral bands at 1024, 1081, and 1620 cm^-1^ and increased bands around 1240 and 1670 cm^-1^ were found in meningiomas MC mal. The Fisher coefficients confirmed the relevance of these spectral regions for WHO grades 2 and 3 (Supplementary Figure 2A) as well as for MC int and MC mal (Supplementary Figure 2B), respectively. Considering IR band assignments,^24^ carbohydrates (C-O vibrations at 1024 cm^-1^) and nucleic acids (symmetric stretching of PO^2-^ at 1080 cm^-1^) might have been reduced in more aggressive types of meningioma phenotype, while (phospho-)lipids (stretching of PO^2-^ at 1240 cm^-1^ and CH_3_ bending at 1450 cm^-1^) were increased. Moreover, the conformation of proteins (beta-sheet band around 1620 cm^-1^, alpha-helix band around 1650 cm^-1^, random coil band around 1670 cm^-1^) was changed. Interestingly, the difference spectra had similar bands except in the Amide I region (1600–1700 cm^-1^; compare Figure 1E and F). The data suggests that there was a change in protein content for the meningiomas WHO grade 2 and 3, while there was rather a change in protein conformation for meningiomas MC int and MC mal.

**Figure 2.**
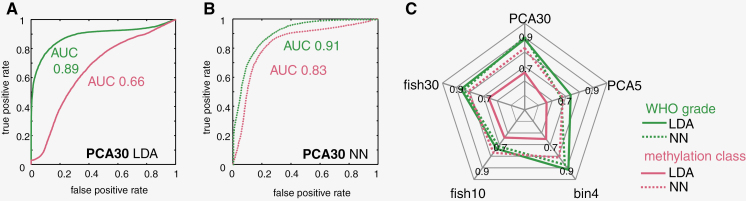
Comparison of different classification approaches for WHO grade and methylation class. Different approaches for data dimensionality reduction were tested followed by either linear discriminant analysis (LDA) or by an optimized neural network (NN). (A) Receiver operating characteristic (ROC) curve for classification of spectra of the test set using the 30 PC and LDA. (B) ROC curve for classification of spectra of the test set using the 30 PC and NN. (C) Spider plot of the area under the ROC curve (AUC) for different combinations of data dimensionality reduction. The type of classifier (LDA or NN) used and the ground truth (WHO grade or methylation class) is indicated by different lines types as shown in the legend.

As we found spectral differences between groups, we then developed classification algorithms. Two different algorithms were developed on respective training sets of IR spectra: one for predicting WHO grade and the other for predicting methylation class for each spectrum using the identical dataset, respectively. Previous studies showed that simple classifiers are well suited to identify brain tumors and extract the WHO grade of glioma.^7,15,25^ Therefore, we first followed established approaches and used PCA for data dimensionality reduction followed by linear discriminant analysis for either WHO grade classification or methylation class analysis (Figure 2A). This strategy was rather successful for WHO grade classification taking into account 30 PCs (AUC 0.89), while it was not suited to identify methylation classes (AUC of 0.66). However, choosing a neural network (NN) classifier drastically improved the classification performance for methylation class analysis (AUC 0.83), while giving comparable results for WHO grade (AUC 0.91, Figure 2B). Interestingly, linear discriminant analysis was generally inadequate to distinguish methylation classes, regardless of the approach for data dimensionality reduction (Figure 2C), whereas results were sufficient for WHO grades. Here, LDA was a sound solution for all conditions tested and the application of an NN did not lead to an improvement of classification.

**Figure 3.**
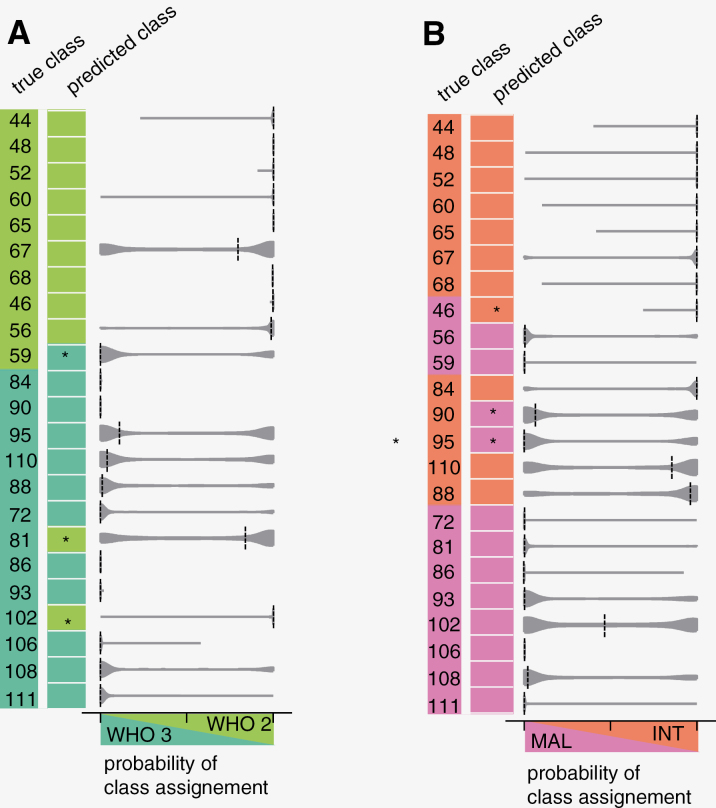
Classification result for each sample of the test set based on the first 30 PC and a neural network classifier. (A) WHO grade and (B) methylation class. The true class (with sample numbers) and predicted class are shown by color code. Misclassified samples are indicated by asterisks. The violin plot shows the probability of class assignment of the spectra for each sample, dashed line indicates the median.

Choosing the classification strategy accordingly (namely, data dimensionality reduction by PCA and NN classification) led to successful prediction of WHO grade (Figure 3A) and methylation class (Figure 3B). For both diagnostic systems, 20/23 samples of the test set were assigned to the correct class by applying a straightforward approach that uses the mean value of class assignments of the spectra as the classification result for a sample. Classification results for each measurement position are shown in Supplementary Figure 4. The distribution of the class assignment probabilities of the spectra is shown as a violin plot in Figure 3. The median of the probabilities showed a clear weighting of a class for almost all the samples, with the exception of sample 102, for prediction of methylation class. Moreover, it becomes clear on first sight that the neural network classifier is kind of “absolutely sure” for about half of the samples (for WHO grade: samples 44, 48, 52, 60, 65, 68, 46, 84, 90, 86, 93, 106; for MC: samples 44, 48, 52, 60, 65, 68, 46, 59, 72, 86, 406, 111). In those cases, almost all spectra of the sample were assigned with probabilities of ~100% to the same class.

Interestingly, samples that were misclassified regarding the WHO grade were correctly classified in terms of methylation class and vice versa (misclassified samples are marked by an asterisk in Figure 3 A, B). This suggests that spectral features associated with the tumor were responsible for the misclassification rather than measurement artifacts or errors. The diagnosis of eight samples of the test set was discordant between WHO grade and MC (samples 46, 56, 59, 84, 90, 95, 110, 88). Interestingly, misclassification for MC occurred only in those cases. Sample 46 (ground truth: MC mal) was misclassified as MC int and had WHO grade 2 and samples 90 and 95 (ground truth: MC int) were misclassified as MC mal and had histopathological WHO grade 3.: WHO.

## Discussion

This study shows that analysis of infrared spectroscopic datasets can provide information on WHO CNS grade and methylation class for meningiomas. It extends previous findings to aggressive meningioma subtypes and relates infrared spectroscopy and diagnostic methylation profiling for the first time.

Vibrational spectroscopy addresses molecular bond vibrations and is sensitive to DNA methylation as shown on isolated chemical compounds.^26^ Although IR spectroscopy can directly visualize methyl groups attached to DNA, this typically necessitates DNA isolation and specialized sample preparation.^27^ In our study, we analyzed tissue sections and the recorded spectra thus comprised information of all cellular compartments. Therefore, the observed spectral changes between meningiomas MC int and MC mal are likely caused by cellular and metabolic remodeling triggered by altered gene expression, rather than from CH_3_ groups bound to the DNA. This is supported by findings of Raman spectroscopy that have previously linked the MGMT methylation status in glioblastoma to metabolic and biochemical changes in lipids.^28^

Changes in similar IR spectral ranges were identified for the respective more aggressive meningioma types in both diagnostic system (WHO grade and MC). Those might be explainable in the context of cellular modifications associated with increasing proliferation and malignancy.^29^ Meningiomas WHO grade 1 and 2 have been investigated using IR spectroscopy before.^19^ The authors identified a reduction of amide I (1650 cm^-1^) band intensity in recurrent WHO 1 meningiomas and WHO 2 meningiomas compared to WHO 1 meningiomas. In our study, a similar observation was made regarding the amide I band that was also reduced in the higher-grade meningiomas. This could be due to the reduction in protein content associated with changes in cell density, fibrous structures, and extracellular fibers. Expression of collagen type V is increased in atypical and anaplastic meningiomas WHO 2/3^30^ and proteomics indicated disturbed collagen biosynthesis and degradation as well as extracellular matrix remodeling in nonskull base meningiomas (WHO 1 and 2).^31^ Moreover, brain invasive meningiomas might strongly express MMP-9 altering extracellular protein.^32^ Interestingly, the change in protein-related band intensity is different in comparison to other types of primary or secondary brain tumors. Here, an increase in protein-related bands together with a decrease in lipid related bands is associated with increasing malignancy.^1,15^ Besides protein modifications, our study suggests changes in carbohydrates, nucleic acids, and (phospho-)lipids. Increased bands related to carbohydrates might be associated with the presence of clear cells with cytoplasmic glycogen deposition, being the name-giving feature of clear cell meningiomas WHO 2.^29^ Moreover, activities of several glycolysis enzymes were altered in anaplastic meningioma compared to benign subtypes.^33^ Likewise, the expression of proteins associated with fatty acid metabolism was linked to meningioma grade and aggressiveness.^34^ Increased bands related to nucleic acids in WHO 2 / MC int seem counter-intuitive, given the increased proliferation (more mitotic figures) that is a key diagnostic feature of WHO 3 meningiomas. It could be nonetheless linked to higher cell density and/or translational activity.

The successful development of a classifier capable of distinguishing between WHO 2 and WHO 3 meningiomas aligns with prior research in the field. In the context of cancer, including brain tumors, IR spectroscopy has been widely utilized, demonstrating its efficacy in classifying specimens based on histological diagnosis.^35–37^ However, there is a considerable degree of heterogeneity among meningiomas even within the various subtypes, which is reflected in the presence of different intratumoral gene and protein expression programs. Here we showed that infrared spectroscopy holds promise for identifying established biomarkers that discriminate between meningioma subtypes or predict clinical outcomes, thereby facilitating more precise diagnosis and prognosis during surgery. Future studies on a larger dataset might extend those findings and employ unsupervised machine learning strategies to further stratify meningiomas and uncover valuable insights into the underlying molecular processes driving tumor progression and response to therapy.

It is important to note that linear classifiers, which are established strategies and that work well for WHO grade, failed in retrieving the MC. The NN classifier exhibited superior performance, presumably because it can better model complex relationships in the data and can potentially capture fine nuances. This is consistent with the general trend that workflows are evolving towards more complex modeling approaches that allow capturing tissue complexity.^35^ In the context of this study, one may speculate that the categorization into MC can be attributed to the complex and interacting processes associated with DNA methylation patterns, while histological WHO grade is based on a more straightforward visual assessment and represents an ordinal type of the grading system. This is supported by the fact that misclassifications for MC occurred only in samples that had a discordant WHO grade. Unfortunately, NN classifiers come with the drawback of being data hungry. While they were well applicable in our ex vivo study that used an IR imaging array for data acquisition and analyzed 35643 spectra, the need for large training sets might be a limitation for studies using fiber-based spectroscopic systems that can only acquire single spectra in one shot. This fact must be considered in the future development of clinical classifiers. Moreover, it might increase the classification accuracy to intentionally focus the training of the algorithm on samples that show conflicting states of malignancy in the two diagnostic systems (ie, MC int WHO3 samples and MC mal WHO 2 samples) to refine the classifier.

Fiber-based instruments offering in situ IR spectroscopy have been made available and have demonstrated large potential for identification of pancreatic cancer.^38^ This implies that intraoperative IR spectroscopy could be likewise performed during meningioma surgery offering immediate diagnostic insights. For this information to be effectively utilized in a clinical setting, algorithms need to be developed and the attending physician must be empowered to rate the output of the classifier. Here, we defined the average of class assignments of all the spectra of one sample as classification result. However, other various strategies for data interpretation exist, presenting additional options to assess the goodness of classification and it may be beneficial to incorporate additional criteria in future clinical applications. One potential approach is to consider the probabilities of class assignments. This could involve setting a classification probability threshold or excluding areas with a high number of spectra classified with low probability. Alternatively, one might evaluate multiple measurement position separately to take into consideration regional variability and determine a conclusive diagnosis for the patient integrating various clinical modalities. These kinds of approach might enhance the overall accuracy and clinical utility, reinforcing the potential of IR spectroscopy in advancing precision medicine for meningioma characterization.

## Supplementary data

Supplementary material is available online at *Neuro-Oncology Advances* (https://academic.oup.com/noa).

vdae082_suppl_Supplementary_Figures
