# Supplementary material for: Prediction of WHO grade and methylation class of aggressive meningiomas: Extraction of diagnostic information from infrared spectroscopic data
Source: Neurooncol Adv. 2024 Jun 6;6(1):vdae082. doi: 10.1093/noajnl/vdae082 (PMC11245706; doi:10.1093/noajnl/vdae082)
Supplement: vdae082_suppl_Supplementary_Figures [file vdae082_suppl_Supplementary_Figures.pdf]

Supporting information

Prediction of WHO grade and methylation class of aggressive meningiomas: Extraction of diagnostic information from infrared spectroscopic data

Roberta Galli, Franz Lehner, Sven Richter, Katrin Kirsche, Matthias Meinhardt, Tareq A. Juratli, Achim Temme, Matthias Kirsch, Rolf Warta, Christel Herold-Mende, Franz L. Ricklefs, Katrin Lamszus, Philipp Sievers, Felix Sahm, Ilker Y. Eyüpoglu, Ortrud Uckermann

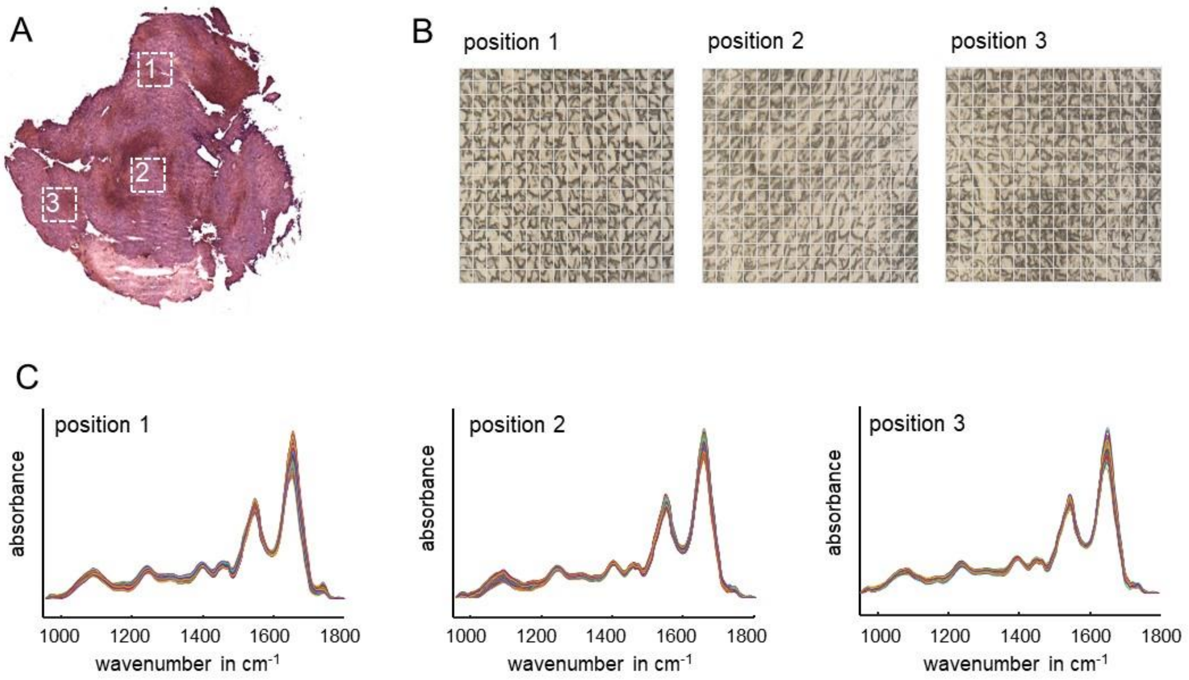

Supporting Figure S1: Acquisition of infrared spectra at three positions of the sample. **A:** Three positions were selected on HE stained tissue section **B:** Selected positions were identified on consecutive unstained tissue sections and infrared spectra were acquired in an area of 170x170 µm. An array of 16x16 spectra was obtained after a 4x4 spatial binning procedure. **C:** Preprocessed IR spectra in the fingerprint region (950-1800 cm^{-1} for each position (each n=256). The example shows a meningioma WHO CNS grade 3, methylation class intermediate-A

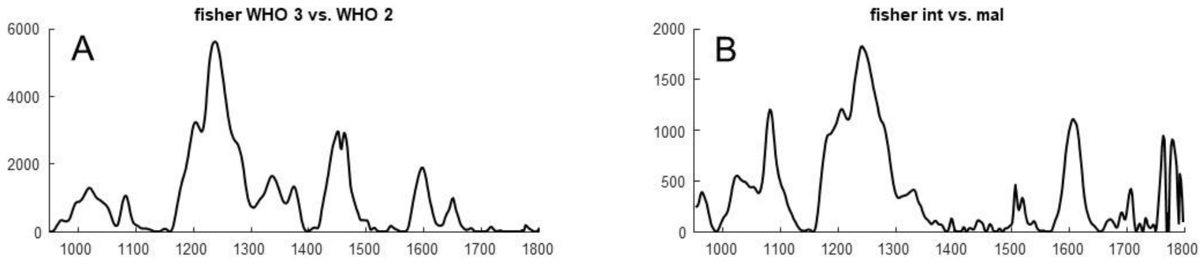

Supporting Figure S2: IR spectral signatures of the training set were analyzed. The Fisher coefficient was calculated to evaluate differences between WHO grade 2 versus WHO grade 3 (A) or methylation class intermediate versus malignant (B).

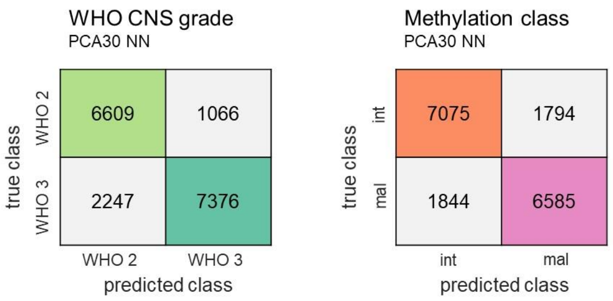

Supporting Figure S3: Confusion matrices for classification of spectra of the test set. The first 30 PCA were used followed by neural network classification

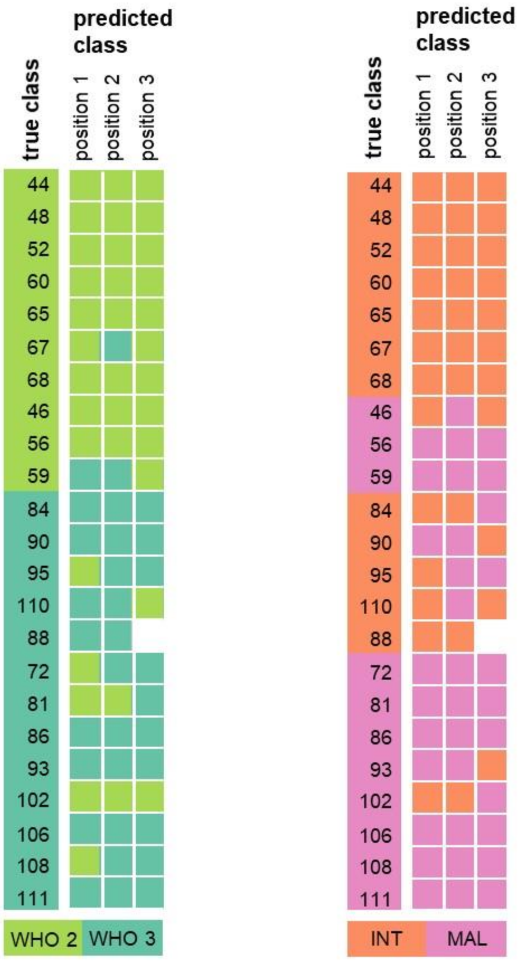

Supporting Figure S4: Classification result for the three measurement positions on each sample (for sample 88 only two positions were measured).
